# Stabilization challenges and aggregation in protein-based therapeutics in the pharmaceutical industry

**DOI:** 10.1039/d3ra06476j

**Published:** 2023-12-11

**Authors:** Mahdie Rahban, Faizan Ahmad, Mieczyslaw A. Piatyszek, Thomas Haertlé, Luciano Saso, Ali Akbar Saboury

**Affiliations:** a Neuroscience Research Center, Institute of Neuropharmacology, Kerman University of Medical Sciences Kerman Iran mrohban@ut.ac.ir; b Department of Biochemistry, School of Chemical & Life Sciences, Jamia Hamdard New Delhi-110062 India faizanahmad@jamiahamdard.ac.in; c Biotechnology Consultant Gainesville FL USA piatyszekm@gmail.com; d National Institute of Agronomic Research Nantes France tom@haertle.fr; e Department of Physiology and Pharmacology “Vittorio Erspamer”, Sapienza University Rome Italy luciano.saso@uniroma1.it; f Institute of Biochemistry and Biophysics, University of Tehran Tehran 1417614335 Iran saboury@ut.ac.ir +9821 66404680 +9821 66956984

## Abstract

Protein-based therapeutics have revolutionized the pharmaceutical industry and become vital components in the development of future therapeutics. They offer several advantages over traditional small molecule drugs, including high affinity, potency and specificity, while demonstrating low toxicity and minimal adverse effects. However, the development and manufacturing processes of protein-based therapeutics presents challenges related to protein folding, purification, stability and immunogenicity that should be addressed. These proteins, like other biological molecules, are prone to chemical and physical instabilities. The stability of protein-based drugs throughout the entire manufacturing, storage and delivery process is essential. The occurrence of structural instability resulting from misfolding, unfolding, and modifications, as well as aggregation, poses a significant risk to the efficacy of these drugs, overshadowing their promising attributes. Gaining insight into structural alterations caused by aggregation and their impact on immunogenicity is vital for the advancement and refinement of protein therapeutics. Hence, in this review, we have discussed some features of protein aggregation during production, formulation and storage as well as stabilization strategies in protein engineering and computational methods to prevent aggregation.

## Abstract 2

In this review, we have discussed some features of protein aggregation during production, formulation and storage as well as stabilization strategies in protein engineering and computational methods to prevent aggregation.

## Graphical abstract

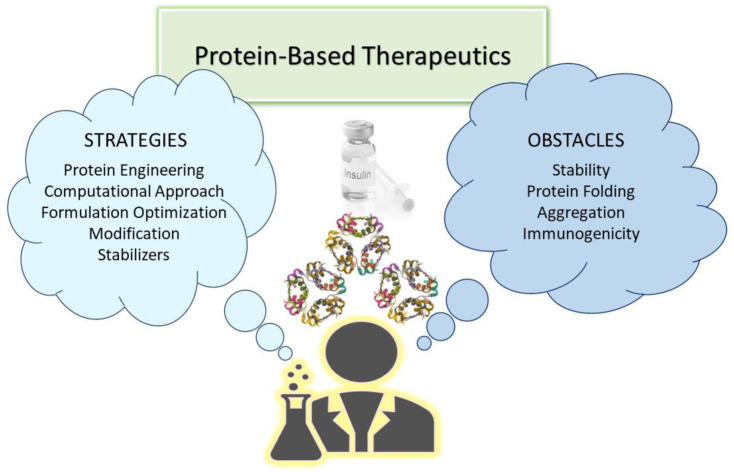

## 1. Introduction

**Fig. 1:**
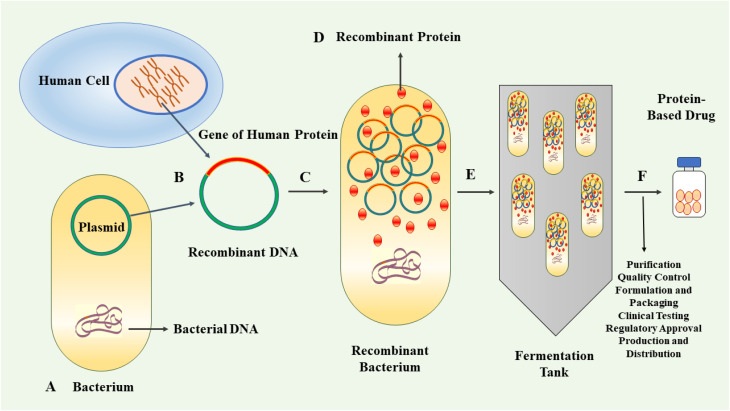
Figure exhibited the process of producing recombinant biopharmaceutical products involves using genetic engineering techniques to create therapeutic proteins. (A) The first step is to select a suitable host organism, often a microorganism like bacteria, yeast, or mammalian cells. The choice of host organism depends on factors such as protein complexity and desired post-translational modifications. (B) The gene encoding the desired therapeutic protein is isolated and cloned into a vector. The vector also contains regulatory elements to control gene expression. (C) In the case of bacteria, the vector with the cloned gene is introduced into the host cells through a process called transformation. (D) Once the gene is inside the host cells, it is transcribed and translated, leading to the production of the therapeutic protein. (E) The cells are then grown in bioreactors (fermentation tanks) under controlled conditions to maximize protein yield. (F) After production, the biopharmaceutical product needs to be separated and purified from the host cell components. This is typically done through a series of chromatography and filtration steps, which isolate the protein of interest. The purified protein undergoes rigorous quality control testing to ensure it meets safety, efficacy, and purity standards. This includes tests for identity, potency, sterility, and absence of contaminants. The purified protein is formulated to the desired concentration and stability. It may also be mixed with excipients to improve shelf-life and administration. Before receiving regulatory approval, the biopharmaceutical product goes through extensive clinical trials to assess its safety and efficacy in humans. Once clinical trials are successful, the product can be submitted for regulatory approval by health authorities. If approved, the biopharmaceutical is produced at a larger scale and distributed to healthcare providers for patient use.

In the last three decades, protein-based therapeutics have emerged as a significant category of pharmaceuticals. Protein-based drugs encompass therapeutic agents consisting of proteins or peptides engineered to interact with specific targets within the body for the treatment or management of diseases.^1–3^ The human proteome contains an estimated 20 000 proteins.^4^ Proteins are highly versatile biomolecules playing crucial roles in various biological processes. They act as catalysts, scaffolds for structural integrity, signalling molecules, molecular transporters and receptors, among other functions.^5,6^ Their versatility enables proteins to contribute to the essential functions and maintenance of cellular and tissue integrity.^7^ Protein-based therapeutics provide a multitude of advantages relative to traditional small-molecule drugs. Notably, their intricate three-dimensional structures confer the ability to interact with specific receptors or molecules in the body with remarkable selectivity and affinity, with minimal side effects.^8–10^ The therapeutic proteins can be derived from natural sources or produced by genetic engineering.^11–13^ The approval and market introduction of human insulin, produced by recombinant technique in *Escherichia coli* (*E. coli*), in 1982, represented an important milestone in the field of protein therapeutics.^14^ Following this achievement, significant progress was made in the cloning and expression of other protein-based drugs, including human growth hormone, interferon β and α, and monoclonal antibodies.^15^ These advancements contributed further to the development of recombinant therapeutics and vaccines.^16^ The production of recombinant proteins has gradually emerged as a vital component within the biopharmaceutical industry.^17,18^ The application of genetic engineering techniques allows for the efficient production of large quantities of target proteins with high consistency and cost-effectiveness compared to traditional methods. This advancement facilitates enhanced availability of therapeutic proteins, thereby improving accessibility for patients requiring these treatments.^19^ By employing recombinant DNA technology, the production of protein-based drugs can be achieved using well-controlled and scalable systems. This allows for the generation of high-quality, consistent and purified protein products.^20,21^Fig. 1 exhibits schematically the process of recombinant biopharmaceutical products.

The progress in genetic engineering technology and a deeper comprehension of diseases provide a promising opportunity in the design and development of new biomolecular entities with desired biopharmaceutical features.^22^ However, several challenges arise in this field. These challenges include: protein aggregation, denaturation, degradation, concomitant loss of activity, immunogenicity, non-specific distribution, rapid clearance from the body and potential toxicity.^23–25^ These issues raise significant concerns that need to be resolved for the successful development and application of protein-based therapeutics.^26^ Recent advances in protein engineering and the ability to intentionally introduce chemical and structural modifications have created a paradigm shift in the fine-tuning of protein properties.^18^ These advancements have enabled researchers to modify proteins at the molecular level, allowing precise control over their functional properties.^27^ This capability has opened new avenues for the design of protein-based therapeutics with enhanced efficacy and stability, optimized pharmacokinetics and reduced immunogenicity. The ability to manipulate and fine-tune protein properties broadens avenues for the development of next-generation biopharmaceuticals.

Protein-based therapy faces various obstacles that can significantly impact its effectiveness and safety. Overcoming these challenges requires a combination of scientific, technological, and regulatory approaches.^17^ Continuous research in protein engineering, advanced drug delivery systems, and manufacturing technologies contributes to addressing these obstacles and enhancing the efficacy and safety of protein-based therapies.^27^Table 1 outlines the specific challenges in protein-based therapy and the corresponding methods to overcome them.

**Table 1:** A summary of the main obstacles in protein-based therapy and the corresponding strategies used to overcome these challenges

Obstacles in protein-based therapy	Strategies to overcome
Protein stability	Formulation optimization, stabilizers, protein engineering for enhanced stability
Immunogenicity	Protein engineering to reduce immunogenic epitopes, immunomodulatory agents
Delivery and targeting	Advanced delivery systems (nanoparticles, liposomes), specific targeting methods
Production costs	Advances in biotechnology, improved manufacturing processes
Dosing precision	Tailoring dosages, controlled-release systems for precision
Regulatory hurdles	Clear documentation, rigorous testing to meet regulatory standards
Short half-life	Modification (*e.g.*, PEGylation) to extend the therapeutic effect, altering formulation
Storage and handling	Proper storage conditions, suitable packaging for preservation

Our aim is to bridge the gap between the broader understanding of protein stability and its specific applications in therapeutic contexts by addressing the obstacles encountered in protein-based therapy due to protein instability.

## 2. Stability challenges of protein-based therapy

**Fig. 2:**
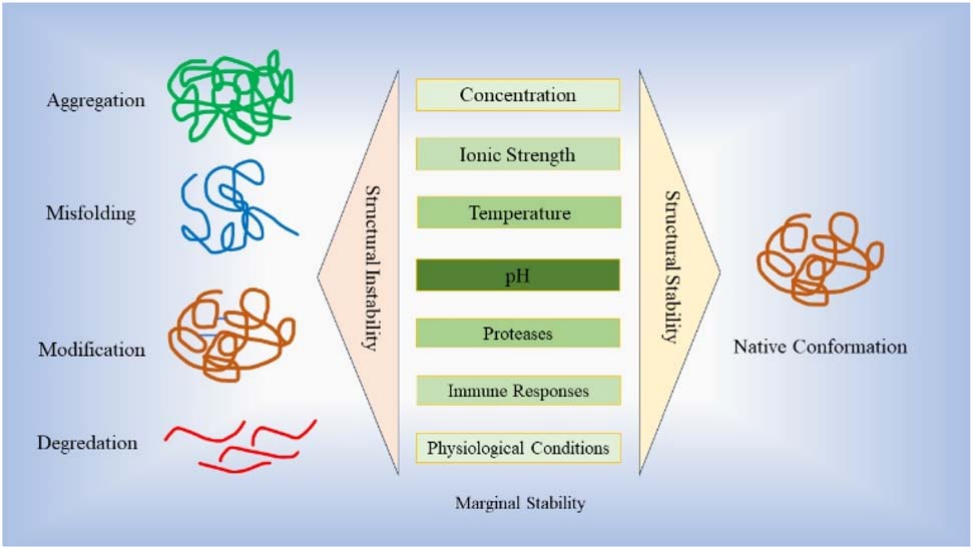
Figure depicts the issues associated with protein-based therapies' stabilities, notably influencing their efficacy and safety attributes. This figure illustrates the critical role of stability in the development of protein-based therapy. Ensuring stability is crucial to maintain the efficacy and functionality of protein-based therapies throughout manufacturing, storage, and delivery processes. Structural instability, arising from misfolding, unfolding, various modifications, and aggregation, can impair drug efficacy. Proteins and peptides exhibit a limited stability range influenced by factors such as concentration, ionic strength, temperature, and pH often referred to as “marginal stability”. Furthermore, proteins face additional challenges caused by proteases, immune responses and human physiological conditions that can impact their stability.

Stability plays a vital role in the development of protein-based therapy, encompassing aspects such as optimal storage conditions and *in vivo* performance.^28^ Proteins are susceptible to various destabilizing factors, such as aggregation, degradation and denaturation, all of which have the potential to downgrade their intended functions.^28,29^ Ensuring stability is essential to preserve the efficacy and functionality of protein-based therapies. The stability of protein-based drugs is of utmost importance during the entire process of manufacturing, storage, and delivery.^30^ Structural instability, which can arise from misfolding, unfolding, and different non-covalent or covalent modifications as well as aggregation, may deteriorate drug efficacy and their promising curative properties.^30,31^ Proteins and peptides demonstrate a restricted range of stabilities, influenced by factors such as concentration, ionic strength, temperature and pH.^32–36^ This narrow range is commonly referred to as “marginal stability”.^37,38^ Moreover, many recombinant proteins are inherently unstable under the conditions in which they are expressed, leading to a loss of correct folding or increased susceptibility to proteolytic digestion. This instability poses a significant challenge to obtaining adequately folded proteins for therapeutic applications.^39^Fig. 2 illustrates the stability hurdles confronting protein-based therapies, with profound implications for their effectiveness, stability, and safety profiles.

Maintaining the appropriate temperature conditions (below the midpoint of denaturation) helps to preserve protein stability and prevent undesirable changes in structure and function over time.^40,41^ Strategies such as cold chain management, using temperature-controlled storage facilities and employing suitable freeze–thaw protocols are employed to ensure the integrity of therapeutic proteins.^41^ Maintenance of proper pH of the environment plays a critical role in preserving the protein structural integrity and prevents undesirable chemical reactions that can lead to degradation.^30^ Due to precise control and optimization of pH conditions, proteins can maintain their native conformations, preserving their biological activities and therapeutic efficacies throughout the entire lifecycle of the product.^42^ The pH significantly influences intramolecular folding and protein–protein interactions by playing a critical role. At various pH values, specific amino acid residues can undergo protonation or deprotonation, thereby impacting their capacity to form hydrogen bonds, form electrostatic and hydrophobic interactions. Alterations in pH can disturb the charge balance within the protein resulting in modifications of its conformation and stability.^37,42,43^ Additionally, certain proteins may interact favourably with surfaces of various vessels, leading to adsorption and decreasing the concentration of the active ingredient available for therapeutic action.^30^ Therefore, a proper formulation encompasses factors such as buffer systems, excipients and stabilizers, which contribute to preserving the protein native structure and preventing undesired modifications. These formulations are designed to create optimal conditions, including appropriate pH, ionic strength and osmolarity, in order to safeguard the stability of therapeutic proteins.^44–46^

Furthermore, by serving as protein folding assistants, chaperones enhance the yield of properly folded proteins, mitigating the formation of inclusion bodies during recombinant protein production.^47^ Inclusion bodies are dense aggregates of misfolded or aggregated proteins that accumulate within the cytosol, nucleus, or periplasm of cells during recombinant protein production.^48^ Integrating chaperones into the production process can significantly improve the overall quality and functionality of the final recombinant protein product.^49^ Molecular chaperones, whether in the cytoplasm or the periplasm, play a crucial role in facilitating the production of properly folded recombinant proteins. In the cytoplasm, chaperones like Hsp100 (Clp), Hsp90, Hsp70 (DnaK/DnaJ/GrpE), Hsp60 (GroEL/GroES), and small heat shock proteins (sHsps) are involved in aiding the correct folding of recombinant proteins.^50^ Periplasmic chaperones in cells are crucial for properly folding recombinant proteins by aiding in disulfide bond formation, ensuring their soluble form.^51^ The efficiency of this process varies based on the protein type and is improved by proteins like FkpA and SurA, aiding in critical peptidyl–prolyl bond isomerization. Moreover, DsbA and DsbC are essential for forming disulfide bridges, preventing misfolded proteins and guiding recombinant proteins toward their functional form in the periplasmic space.^50^ Certain chemical additives in culture media enhance the expression of soluble recombinant proteins by inducing osmotic stress and triggering the production of osmolytes like betaine and trehalose in *E. coli* cells. These osmolytes serve as “chemical chaperones,” boosting the stability of native proteins and aiding in refolding unfolded polypeptides. Notably, d-sorbitol, in combination with betaine, is widely employed to reduce the formation of inclusion bodies in recombinant proteins.^52^

In the complex environment of the human body, proteins face additional challenges caused by proteases, immune responses and physiological conditions that can impact their stability. Understanding and addressing these stability concerns are critical for ensuring the effectiveness and safety of protein therapeutics.^53,54^ To address these stability concerns, various structural modifications such as site-specific mutations, post transcriptional modifications (PTMs) and PEGylation, have been explored to improve protein solubility and stability. These modifications aim to enhance the overall performance and longevity of protein therapeutics.^55–59^

In addition to stability, protein-based therapeutics need to possess suitable pharmacokinetic and pharmacodynamic properties to ensure optimal functionality.^60^ Pharmacokinetics encompasses absorption, distribution, metabolism and excretion of the therapeutic proteins within the body. It is important for the protein to exhibit appropriate bioavailability, distribution in the target site and elimination from the body.^61^ On the other hand, pharmacodynamics relates to how the proteins interact with their targets and produce desired therapeutic effects. This includes factors such as binding affinity, receptor activation and downstream signalling pathways.^25,60^ Achieving the desired pharmacokinetic and pharmacodynamic profiles is crucial for the efficacy, safety and dosing regimen optimization of protein-based therapeutics. It requires careful design, optimization and characterization of the protein molecules, to ensure that they properly interact well with the therapeutic target and achieve the desired therapeutic effects with none or negligible off-target effects.^25,60,62–64^

### 2.1 Immunogenicity and protein aggregation

One of the primary challenges associated with the production, distribution and storage of protein-based drugs is the risk of protein aggregation. Aggregation refers to the process in which proteins associate and form clusters or aggregates, which can negatively impact the therapeutic efficacy.^65^ Aggregated proteins may be recognized by the immune system as foreign or abnormal, triggering an immune response that can result in the production of antibodies against the therapeutic protein.^66^ The immunogenic potential of all aggregate types remains uncertain and subject to debate within the scientific community. It is not yet fully understood whether all aggregates have the potential to elicit an immune response. Furthermore, the impact of additional clinical factors on immunogenicity is a complex area requiring further investigation.^67,68^ Immunogenicity refers to the development of serum anti-drug antibodies (ADAs) that specifically target and bind to the proteins of interest, such as a therapeutic proteins or drugs. This immune response can have significant implications for the efficacy and safety of the protein products and can lead to reduced their effectiveness.^69,70^ When ADAs bind to the therapeutic protein, they can neutralize its activity, prevent it from reaching its target or accelerate its clearance from the body. This can result in diminished therapeutic effects and reduced clinical benefits to the patients. In more severe cases, the formation of ADAs can pose serious health concerns.^71,72^ The immune response triggered by ADAs can lead to adverse immune reactions, including hypersensitivity reactions, allergic responses or immune-mediated diseases. Examples include the formation of ADAs leading to reduced efficacy or life-threatening conditions like pure red cell aplasia (PRCA) with Eprex.^73^ Thorlaksen *et al.*^74^ showed that various aggregate populations are more immunogenic compared to the native human insulin, and the degree of immune activation depends on specific aggregate features.

**Fig. 3:**
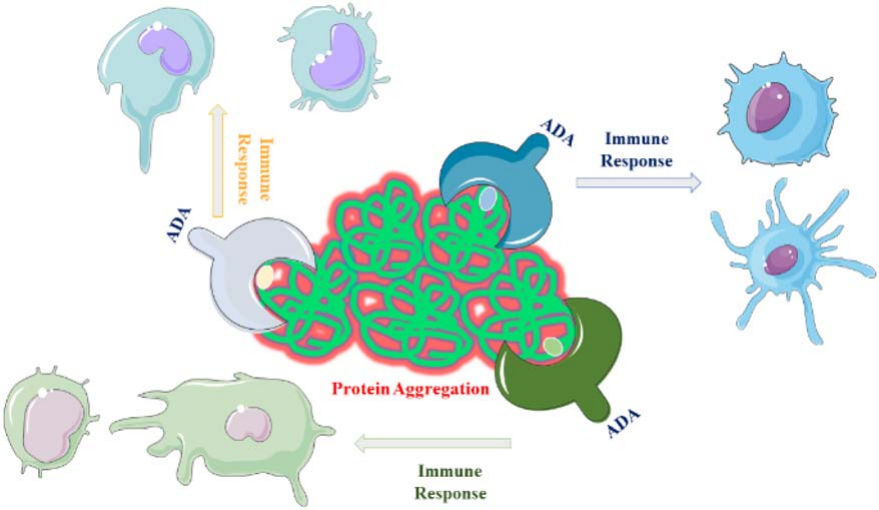
The illustration depicts how protein aggregates can enhance the immunogenicity of a therapeutic protein by triggering the formation of serum anti-drug antibodies (ADAs) that bind to the protein. The distinct aggregate types activate different immunological pathways.

Aggregates containing flexible, micron-sized particles with altered secondary structure were found to be highly immunogenic.^75^ Compact micron-sized particles with native-like structures also exhibited immunogenicity *in vivo*. Subvisible were more immunogenic than submicron particles or soluble oligomers^76^ which, is discussed in the next section. Chemical modifications of the insulin molecule did not significantly impact immunogenicity.^77^ The correlation between *in vitro* and *in vivo* immunogenicity assessments suggests that distinct aggregate types activate different immunological pathways, contributing to overall immunogenicity.^74^ A study demonstrated that changes in protein structure resulting from aggregation can alter the range of antigens targeted by the immune response. Protein aggregation increases the number of epitopes presented to major histocompatibility complex class II (MHC II) molecules.^78^ For example, an assay comparing non-aggregated and aggregated formulations of two human IgGs subjected to heat and shake-induced aggregation demonstrated a significant increase in the number of epitopes presented on monocyte-derived dendritic cells (MoDCs).^23^ This suggests that structural changes induced by aggregation may cause immune responses targeting antigens that are chemically modified or hidden in the native fold of the proteins (Fig. 3). This hypothesis is further supported by research using humanized single-chain variable antibody fragments (scFv).^23,79^

Peptide mapping and enzyme-linked immunosorbent assay (ELISA) identified a peptide sequence, which is highly targeted by antibodies induced by protein aggregates but not by monomers in wild-type mice.^23^ Molecular dynamics (MD) simulations, protein structure prediction software and simulated annealing revealed that this peptide sequence was located in a hydrophobic domain of the scFv, which tends to become exposed due to partial unfolding.^80^ Additionally, while native aggregates increased anti-drug antibody (ADA) levels, non-native aggregates induced ADA levels similar to, or lower than those induced by the monomer.^23^ This suggests that the native conformation of the protein is important for immunogenicity. Understanding the structural changes induced by aggregation and their effects on immunogenicity is crucial for the development and optimization of protein therapeutics.^23,80^

Analytical techniques and immunogenicity assessments are utilized to detect and evaluate protein aggregates. Monitoring and managing immunogenicity is crucial, and it involves preclinical and clinical testing, assay development, and strategies to minimize immune responses. By addressing immunogenicity, the efficacy and safety of protein products can be optimized for better therapeutic outcomes.^81,82^

## 3. Aggregation in protein-based therapeutic products

Proteins strive to maintain their native conformations, driven by thermodynamics and the lowest energy states.^83^ In the folded protein, hydrophobic amino acid residues are buried in the protein avoiding contact with water.^84^ However, it is important to note that not all hydrophobic residues are buried within the protein structure. Water molecules can form loose structures (cages) around exposed hydrophobic regions. Consequently, folding, surface adsorption or collapse of hydrophobic patches in proteins can result in the release of structured (caged) water into the surrounding solvent, thereby increasing the overall system entropy.^29,85,86^ Hydrophilic residues (uncharged and charged amino acids) would prefer to be on the surface of the folded protein. In fact, burial of charged side chains is very rare.^87^ In contrast, buried uncharged polar side chains are very common. These buried uncharged polar side chains are involved in hydrogen bond formation; it is very rare to find unpaired hydrogen bond donors and acceptors. These buried hydrogen bonds are more stable than those exposed to polar solvent water. In addition to hydrophobic interactions and hydrogen bonds, other noncovalent interactions that contribute to the thermodynamic stability are van der Waals interactions and salt bridges.^88^

Monomeric folded proteins are marginally stable. This low thermodynamic stability may facilitate conformational changes within the protein structure, which can ultimately lead to protein instability and the subsequent aggregation.^85^ Protein aggregation is a complex process that can occur through three main mechanisms, depending on the seeding entity involved, *i.e.*, the native monomers, denatured proteins and pre-existing aggregates.^89,90^ Two or more native monomers, which exist in folded state, can self-associate and form oligomers. This self-association can be driven by complementary charge–charge interactions between different monomers or through the formation of noncovalent linkages between hydrophilic and hydrophobic residues on the protein surface. These interactions can lead to the assembly of larger aggregates.^91^ The size of aggregates increases over time and they become more resistant to get back to their native states. It is commonly observed that protein products contain a fraction of denatured proteins, which have a higher propensity for irreversible aggregation. While partially unfolded proteins may possess the ability to refold under specific conditions, the thermodynamics and kinetics generally favour the aggregation process over refolding.^92–94^ Another mechanism is that the native protein monomers can aggregate by adhering to pre-existing protein oligomers, contaminants or to the surfaces of containers. This adherence can initiate a nucleation process in which the aggregates rapidly grow in size. This expansion of aggregates further contributes to protein aggregation.^30^

Soluble aggregates have low molecular mass and may exhibit reversibility, while insoluble aggregates exceed the solution solubility limit and precipitate out of the solution.^69^ In biological products, a small amount of soluble aggregates (5–10%) is generally considered acceptable. Insoluble aggregates are evaluated based on the size of particles detected upon reconstitution. Particles as small as 150 μm in diameter can be visually detected in injectable products and particles below 150 μm are more likely to induce immune reactions. Subvisible particles with a hydrodynamic radius of ≤50–100 μm are deemed acceptable, while particles ≥10 μm can obstruct blood flow. The U.S. Pharmacopeia (USP) sets the limit for particulate matter in a container of ≤100 mL to be 6000 particles ≥10 μm and 600 particles ≥25 μm. However, recent regulatory scrutiny by the U.S. Food and Drug Administration (FDA) has emphasized the importance of controlling aggregates below 10 μm in biological products. Aggregates of this size pose a greater risk to product stability and immunogenicity.^76,85^

Colloidal stability of proteins relies on the intermolecular interactions among protein molecules. These interactions involve various forces, including short-range attractions, steric repulsions and electrostatic interactions. Short-range attractions arise from van der Waals forces between protein molecules when they come within a certain distance of each other. Steric repulsion occurs when protein molecules approach each other closely, leading to repulsive forces due to overlapping electron clouds or steric hindrance.^95^ Electrostatic interactions involve the attraction or repulsion of charged protein molecules based on their electrostatic charges. These intermolecular forces play a crucial role in determining the colloidal stabilities of proteins.^96^ The balance between these forces can either promote or hinder protein aggregation. For example, a strong steric repulsion and electrostatic repulsion between protein molecules can prevent their close association and aggregation.^97^ Otherwise, if the attractive forces outweigh the repulsive forces, protein molecules may associate and form aggregates. Therefore, understanding and manipulating these intermolecular interactions are important in controlling the colloidal stability of proteins.^98^

Aggregate morphology, influenced by the protein and stress conditions, plays a crucial role in the surface area and subsequent immunogenicity of aggregates. Comparisons between globular and filamentous aggregates of human IgG models revealed that filamentous aggregates induced higher expression of immune activation markers (CD86, CD80, CD83) on MoDCs. Filamentous aggregates were predominantly formed under heat and shaking stress, while shear stress or freeze–thawing induced more globular aggregates.^23,78^ Significantly, it has been observed that globular aggregates have a lower surface area relative to their mass/volume ratio compared to elongated filamentous aggregates. This observation implies that an increased surface area could potentially contribute to enhanced immune activation.^99^

In addition, oxidation induced by UV light exposure or metal contamination as well as temperature, pH, ionic conditions, mechanical agitation and freeze–thawing pressure, are among the key factors that can impact protein aggregation.^100–104^ The pH of a solution has been found to exert diverse effects on protein aggregation. Extreme pH conditions or significant pH shifts from the protein isoelectric point (pI) can disrupt the electrostatic interactions and hydrogen bonds within protein. This disruption often results in a decrease in protein solubility accompanied by conformational changes that ultimately lead to aggregation.^105–107^ Moreover, alterations in ionic conditions of solvents such as changes in the concentration of salts or divalent cations, can also influence protein solubility and facilitate aggregation.^108^ For instance, in the context of monoclonal antibodies (mAbs), a recent study indicated that mAbs formulated in a sodium acetate buffer demonstrated more conformational and colloidal stabilities compared to those formulated in a sodium citrate buffer.^109,110^

Protein and peptide aggregation depends on protein concentration, and the presence of metal ions can affect the rate of aggregation. However, the relationship between metal ions and protein aggregation is complex and not fully understood. Under normal physiological conditions, aggregation typically does not occur, but it can be induced by certain metal ions.^111,112^ Metal ions like Zn^2+^, Cu^2+^ and Pb^2+^ can inhibit or accelerate aggregation. Overall, understanding the precise impact of cations on protein aggregation remains a challenge, and further research is needed to establish a consensus.^37,111,113,114^

Chemical modifications, such as deamidation and oxidation, can have a significant impact on the stability of protein-based therapeutics, including antibodies. Deamidation involves the conversion of asparagine to aspartic acid or isoaspartic acid, while oxidation involves the addition of oxygen or reactive oxygen species to amino acid residues. Both modifications can lead to conformational changes, reduce solubility and increased aggregation of antibodies. These modifications can occur spontaneously or be induced by various factors.^115,116^ In a study by Alam *et al.*,^116^ it was demonstrated that methionine oxidation in antibodies leads to a significant reduction in conformational stability. However, this oxidation has a limited effect on antibody aggregation, except under extreme conditions such as low pH and elevated temperature. Conversely, tryptophan oxidation and asparagine deamidation have minimal impact on antibody conformational stability but promote aggregation over the broader range of solution conditions.^116^

Furthermore, various factors contribute to protein aggregation. Mechanical agitation such as stirring, pumping or filtration, can disrupt protein structures and facilitate aggregation by inducing unfolding and exposing aggregation-prone regions. Freeze–thaw cycles encountered during storage, can exert pressure on proteins, resulting in conformational changes, unfolding and aggregation.^69,117^ Exposure to ultraviolet (UV) light can trigger chemical reactions and oxidation, altering protein structures and promoting aggregation. Temperature plays a crucial role as elevated temperatures enhance molecular motions and unfold proteins, whereas extremely low temperatures can induce conformational changes and increase the risk of aggregation.^91^

## 4. Stabilization strategies to prevent aggregation of protein-based therapy

**Fig. 4:**
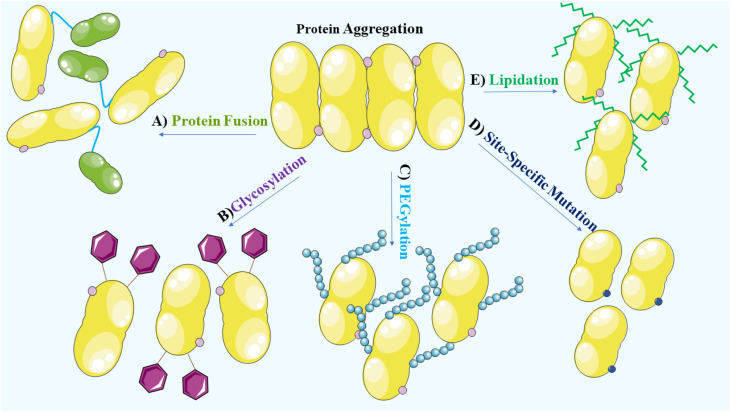
A schematic representation demonstrates the utilization of various strategies to enhance the solubility and stability of protein aggregates, vital considerations in the development of protein-based therapy. These strategies include: (A) protein fusion, (B) glycosylation, (C) PEGylation, (D) structural modifications and site-specific mutation, and (E) lipidation.

To prevent aggregation of proteins, it is crucial to consider and manage the environmental factors during the manufacturing and formulation development processes. During the manufacturing, processing, storage and delivery of protein therapeutics, various stresses can arise leading to protein aggregation and the occurrence of covalent modifications. These modifications encompass oxidation, deamidation, formation of disulfide bridges and cross-linking, which alter the native conformation of proteins.^27^ Methionine, cysteine, histidine, tryptophan, tyrosine and phenylalanine are vulnerable to oxidation and promote aggregation.^23,118^ In addition, deamidation of asparagine and glutamine, disulfide bridge formation involving two cysteine residues and cross-linking due to the formation of covalent bonds between protein molecules, contribute to the aggregate formation. Consideration of these factors is, therefore, vital for maintaining the stability and effectiveness of protein therapeutics.^27^ Structural modifications, site-specific mutations,^27^ glycosylation,^119^ PEGylation,^120^ lipidation^121^ and protein fusion^122,123^ that are widely used to enhance the solubility and stability of proteins, are vital considerations in the development of protein-based therapeutics (Fig. 4).

PEGylation is a widely employed method to enhance the stability of biotherapeutics. It involves the covalent attachment of polyethylene glycol (PEG) chains to the target protein.^124,125^ PEGylation has significant pharmacokinetic advantages that include prolonged circulation time and reduced clearance. The addition of PEG increases the hydrodynamic size of the protein, resulting in decreased renal filtration and an extended half-life. This leads to improved therapeutic efficacy. PEGylation also provides a steric barrier that protects the protein from proteolytic degradation and immune recognition. Furthermore, it improves solubility and stability, preventing aggregation and denaturation under various environmental conditions.^56,126^ The selection of an appropriate PEGylation strategy, including site of attachment and PEG chain length, is essential for achieving desired stability enhancements. Care must be taken to balance the benefits of PEGylation with potential drawbacks, such as altered protein activity and potential immunogenicity of the PEGylated biotherapeutics.^124,127^

Optimizing the freeze-drying or lyophilization process and formulation parameters is crucial for minimizing aggregation. The choice of freezing procedure should consider the specific protein stability requirements to prevent conformational changes and subsequent aggregation, ultimately reducing the risk of immunogenicity.^128^ During the freezing step in lyophilization, the solution is cooled to initiate ice nucleation and the growth of ice crystals. The freezing temperature is selected below the formulation glass transition temperature, and the freezing rate is chosen based on the desired ice crystal size and protein stability. Sufficient holding time at the freezing temperatures ensures thermal equilibrium. An annealing step can follow freezing to promote ice crystal growth and excipient crystallization. Annealing temperature is typically above the glass transition temperature but below the eutectic melting temperature.^129,130^ Optimization of these parameters is essential for achieving efficient drying and uniform freeze-dried products.^131^ Stabilizers play a crucial role in freeze-dried protein formulations to protect proteins during the freezing process. Cryoprotectants such as sucrose and trehalose are commonly used stabilizers. These cryoprotectants work by selectively excluding themselves from the protein surface, thereby raising the energy barrier for protein unfolding during freezing. This mechanism preserves effectively the native state of proteins, enhancing their stability throughout the freeze-drying process.^132–134^ Furthermore, proteins tend to adsorb at different interfaces, including the air–liquid interface during mixing or the ice–liquid or ice–air interface during freeze-drying. To counteract this phenomenon and enhance protein stability in such scenarios, surfactants are incorporated into protein formulations. Non-ionic surfactants such as Polysorbate 20, Polysorbate 80 and Poloxamer 188 are frequently employed in both liquid and freeze-dried protein formulations. These surfactants assist in preventing protein aggregation and adsorption at interfaces, thereby preserving the integrity and functionality of the proteins throughout the formulation and manufacturing processes.^133,135^ A study of a model protein, myoglobin, known to be susceptible to cold denaturation, emphasized the significance of employing a fast-freezing technique to mitigate aggregation and maintain the activity. The hypothesis proposed that rapid freezing reduces the exposure of the protein to cold conditions, where denaturation can occur. However, this fast freezing approach leads to the generation of small ice crystals, which, in turn, enhances the surface area available for interactions between ice and water molecules.^128^ Although the fast-freezing procedure proves beneficial for proteins prone to surface-induced conformational instability, it may not be suitable for all proteins. To illustrate this point, lactate dehydrogenase, another model protein, was employed as an example. The study demonstrated that the same fast-freezing method that effectively preserved myoglobin stability had adverse effects on lactate dehydrogenase. It led to protein aggregation and a subsequent loss of enzymatic activity.^136^ This study emphasizes the significant variation in protein stabilities and the importance of adjusting environmental parameters accordingly to prevent potentially immunogenic aggregate formation.

## 5. Protein engineering strategy

### 5.1 Site-specific mutation

Protein engineering is a crucial component of protein-based therapy, enabling the creation of tailored proteins with improved therapeutic properties. Through computational modelling and structural analysis, protein engineers modify amino acid sequences, introduce site-specific mutations and incorporate functional motifs to optimize protein stability, binding affinity and enzymatic activity.^137,138^ Furthermore, protein engineers have the ability to introduce or manipulate post-translational modifications (PTMs) in therapeutic proteins. PTMs such as glycosylation, phosphorylation, or lipidation exert substantial effects on protein stability, pharmacokinetics and immune recognition. These protein engineering strategies enhance the therapeutic potential of proteins and contribute to the development of more effective protein-based therapies.^139,140^

Site-specific mutations involve targeted alterations to specific amino acids within a protein sequence, exerting significant effects on its properties including solubility and stability.^141^ These mutations are designed to address issues such as protein aggregation, susceptibility to proteolytic degradation and unfavourable interactions with container surfaces. By strategically introducing mutations at precise locations, researchers can disrupt aggregation-prone regions, enhance resistance to proteolysis and mitigate undesirable adsorption, thereby improving the protein solubility and stability.^27^

Here are some specific examples of insulin variants that have been developed through site-specific mutagenesis to achieve different kinetics of action.

• Rapid-acting insulin analogues such as lispro (Humalog): lispro was developed through site-specific mutagenesis by exchange of proline and lysine amino acids at positions 28 and 29 in the insulin B chain. This modification enables lispro to be quickly absorbed after subcutaneous injection, leading to a faster onset of action than regular human insulin. Consequently, lispro closely imitates the rapid insulin secretion observed after meals, offering improved postprandial glucose control for individuals with diabetes.^142^

• Long-acting insulin analogues (Lantus, Toujeo): insulin glargine was engineered through site-specific mutagenesis by replacing asparagine at position 21 of the insulin A chain with glycine and adding two arginine residues at the C-terminus of the B chain. These modifications influence the solubility and stability of the insulin molecule, leading to the formation of micro-precipitates following injection. These micro-precipitates enable a gradual release of insulin over an extended duration, providing basal insulin coverage for up to 24 hours.^143,144^

### 5.2 Computational methods

**Fig. 5:**
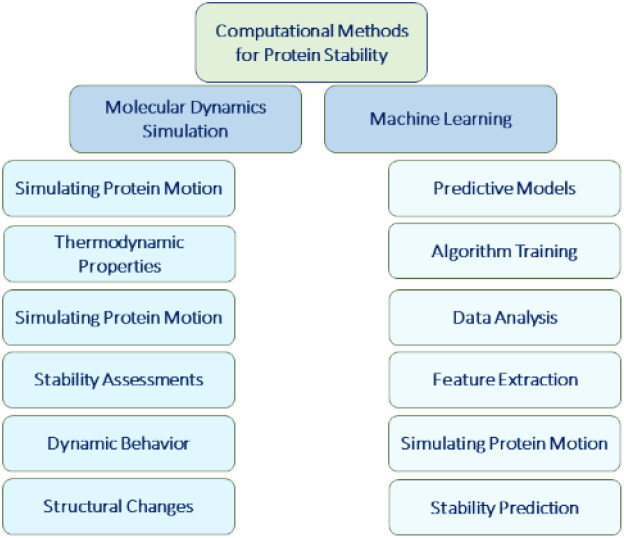
This diagram represents primary branches of computational methods used in protein stability prediction.

The combination of computational methods and experimental approaches accelerates the development and optimization of protein-based therapy by offering valuable insights into protein structure, dynamics, interactions and design. These methods contribute to the rational design of therapeutic proteins, identification of potential drug candidates and better understanding of the underlying mechanisms of protein function and disease.^145–147^ Computational methods such as molecular dynamics (MD) simulations and machine learning algorithms play a crucial role in predicting protein stability for protein-based therapy. These techniques analyse protein structures and sequences to identify regions that are prone to instability.^148^ Furthermore, they can predict the impact of mutations or modifications on protein stability, facilitating the design of more stable protein variants.^84,149^ These computational methods provide valuable insights into protein stability and contribute to the development of more robust and effective protein-based therapies. MD simulations are computational tools that simulate the movement of atoms and molecules over time. In the protein-based drugs, MD simulations provide insights into protein dynamics, stability and behaviour.^150^ They assess protein stability under various conditions and predict how proteins will behave. MD simulations also offer a way to study protein-ligand interactions, helping researchers to understand the binding affinities and to optimize drug candidates.^151–153^ Moreover, machine learning algorithms play a pivotal role in the protein-based therapy by leveraging large datasets of protein stability information to train predictive models. These models analyse protein sequences, structures and other pertinent features, enabling accurate predictions of protein stability.^154–156^ By harnessing the power of machine learning, researchers can streamline the screening and prioritization of protein candidates based on their predicted stabilities. This approach minimizes the need for laborious and expensive experimental screening methods, allowing researchers to allocate resources toward the most promising candidates for further development. Machine learning expedites the identification and optimization of stable protein candidates, facilitating drug discovery of protein-based therapeutics (Fig. 5).^157,158^

### 5.3 Rational design

Rational protein engineering is an approach utilized to enhance protein stabilities by strategically introducing targeted modifications based on a thorough understanding of the protein properties.^159^ Through a comprehensive analysis of the protein structure, dynamics and interactions, specific amino acid substitutions or structural modifications can be implemented to improve stability.^84^ Computational tools, such as molecular modelling and energy calculations, are instrumental in guiding these rational design efforts.^160^ By utilizing these computational tools, researchers can make rational decisions on which modifications are the most likely to enhance protein stabilities, leading ultimately to the development of more robust and effective proteins for protein-based therapeutics.^161^

Deep learning is applied to unlabelled amino acid sequences to extract essential protein features in rational protein engineering. Alley *et al.*^162^ used unsupervised learning with a recurrent artificial neural network called UniRep (Unified Representation). This model was trained on the UniRef50 dataset, which consists of 24 million amino acid sequences. UniRep generates fixed-length vectors that summarize the information in protein sequences, independent of structural or evolutionary data. These vectors contain rich information and have excellent generalization ability, making them suitable for protein stability prediction. UniRep also significantly improves efficiency of protein engineering tasks, making it a versatile tool for protein engineering informatics.^162^

Wang *et al.*^163^ developed an innovative computational method named BayeStab, specifically designed for predicting changes in protein thermostability caused by mutations. This method serves as a valuable tool for researchers evaluating the impact of amino acid substitutions on protein stability. By utilizing BayeStab, scientists can make accurate predictions regarding the thermodynamic stability of mutated proteins, facilitating the rational design and optimization of proteins for various applications in protein-based therapeutics.^163^

Furthermore, the development of DeepDDG predicts changes in protein stability caused by point mutations. By utilizing neural network-based techniques and comprehensive training on a diverse dataset, DeepDDG enhances the ability to understand and manipulate protein stability, facilitating protein engineering efforts and advancing the understanding of missense mutations in proteins. Input features of DeepDDG demonstrated that the solvent-accessible surface area (SASA) of the mutated residue is the main character for the determination of protein stability.^164^

## 6. Prediction of protein aggregation by computational tools

Successful prediction of protein aggregation requires the adaption of computational schemes tailored to the specific properties of the protein being studied. However, this task can be challenging for untrained users due to the requirement of in-depth knowledge about the available computational tools. Understanding the intricacies of these tools is crucial for effectively applying them to analyze protein aggregation. SAP (Spatial Aggregation Propensity) is a technology used to identify regions within therapeutic antibodies that are prone to aggregation. By employing full antibody atomistic molecular dynamics simulations, researchers can pinpoint these aggregation-prone regions. This knowledge allows them to perform targeted mutations and engineer antibodies with improved stability. The application of SAP has significantly enhanced in the stability of therapeutic antibodies compared to the wild type. This technology has the potential to be integrated into the screening and discovery phase of antibody development, enabling the development of more stable and effective antibodies for different diseases.^165^

In a study by De Baets *et al.*, a comprehensive analysis was performed on 611 protein sequences and their respective lifetimes using the aggregation predictor known as TANGO. The results of the study revealed an interesting correlation between protein lifetime and aggregation propensity. It was observed that proteins with shorter lifetimes exhibited a higher tolerance for aggregation.^166^ Phenomenological algorithms utilize experimental data to understand the factors that contribute to protein aggregation and establish empirical aggregation scales. These algorithms, such as AGGRESCAN^167^ and Zyggregator,^168^ are based on the rationalization of experimentally determined factors that influence protein aggregation. Incorporating these factors allows the algorithms, to predict the protein aggregation, more effectively.^169^

The second class of computational approaches is centered around the theoretical assessment of specific sequence properties associated with protein aggregation. Algorithms within this category such as TANGO,^166^ PASTA 2.0,^170^ Amyloid Mutants,^171^ FoldAmyloid^172^ and Waltz^173^ analyze various characteristics of protein sequences. These include the propensity of a sequence to adopt a defined β-enriched conformation, the packing density of proteins, the composition and patterning of residues and the ability to adopt topologically restricted conformations commonly observed in amyloid-like states. By considering these sequence properties, these algorithms offer insights into the potential for protein aggregation, enabling researchers to predict and understand aggregation tendencies more effectively.^145^

Studies of protein aggregation are increasingly relying on the development of machine learning methods. These methods harness the capabilities of artificial neural networks to detect sequential features that exhibit a strong correlation with protein aggregation. Remarkably, machine learning approaches have achieved comparable, and in some cases even superior, performance compared to the traditional predictors.^174^ Algorithms such as APPNN,^175^ FISH Amyloid^176^ and netCSSP^177^ are noteworthy examples of these machine learning-based approaches. These algorithms effectively utilize the power of neural networks to accurately identify and predict protein aggregation, offering researchers valuable tools for studying and understanding this important phenomenon.

## 7. Conclusions

The stability of protein-based drugs is a crucial factor in ensuring their functional integrity for therapeutic applications. Challenges related to proper conditions during manufacturing, storage and transportation as well as immune responses, proteases and physiological conditions in the human body, significantly influence protein stabilities. Protein aggregation poses a substantial risk to therapeutic efficacy as it can induce immunogenicity and diminish the effectiveness of protein products. It is important to note that the choice of stabilization strategies may depend on the specific characteristics of the protein-based drugs, including its size, complexity and intended application. Each protein may require a tailored approach to optimize stability and ensure its therapeutic efficacy and safety. The integration of multiple computational and experimental methods is necessary to address the various challenges in protein-based therapeutic development. In this context, computational methodologies provide value by accelerating experimental processes for design and optimization of therapeutic proteins. Furthermore, this integration of computational and experimental methods provides a more efficient way of reaching a deeper understanding of drug discovery processes and their Overall, the combined efforts of computational modeling and experimental approaches hold promise in advancing therapeutic protein design and implementation in clinical settings.

## Author contributions

The manuscript's evolution was a collaborative effort among the authors. Mahdie Rahban initially drafted the manuscript based on insights received from Ali Akbar Saboury. Faizan Ahmad meticulously reviewed the first draft, offering insightful comments for improvement. Subsequently, Mieczyslaw A. Piatyszek, Thomas Haertlé, and Luciano Saso independently reviewed the manuscript, providing valuable corrections and comments. Finally, Mahdie Rahban and Ali Akbar Saboury amalgamated all the corrections and comments from the co-authors, shaping the final manuscript. This version underwent confirmation and approval by all authors before submission.

## Conflicts of interest

There are no conflicts to declare.

## Supplementary Material
